# Sizing the association between lifestyle behaviours and fatness in a large, heterogeneous sample of youth of multiple ethnicities from 4 countries

**DOI:** 10.1186/1479-5868-10-115

**Published:** 2013-10-12

**Authors:** John D Sluyter, Robert KR Scragg, Lindsay D Plank, Gade D Waqa, Kalesita F Fotu, Boyd A Swinburn

**Affiliations:** 1 Pacific Health, School of Population Health, University of Auckland, Auckland, New Zealand; 2 Epidemiology and Biostatistics, School of Population Health, University of Auckland, Auckland, New Zealand; 3 Department of Surgery, School of Medicine, University of Auckland, Auckland, New Zealand; 4 Fiji School of Medicine, College of Medicine, Nursing and Health Sciences, Fiji National University, Suva, Fiji; 5 Tonga Health Systems Support Program, Ministry of Health, Nuku’alofa, Tonga; 6 WHO Collaborating Centre for Obesity Prevention, Deakin University, Melbourne, Australia

**Keywords:** Television, Soft drink, Breakfast, Physical activity, Obesity, Meta-analysis

## Abstract

**Background:**

The magnitude of the relationship between lifestyle risk factors for obesity and adiposity is not clear. The aim of this study was to clarify this in order to determine the level of importance of lifestyle factors in obesity aetiology.

**Methods:**

A cross-sectional analysis was carried out on data on youth who were not trying to change weight (n = 5714), aged 12 to 22 years and from 8 ethnic groups living in New Zealand, Australia, Fiji and Tonga. Demographic and lifestyle data were measured by questionnaires. Fatness was measured by body mass index (BMI), BMI z-score and bioimpedance analysis, which was used to estimate percent body fat and total fat mass (TFM). Associations between lifestyle and body composition variables were examined using linear regression and forest plots.

**Results:**

TV watching was positively related to fatness in a dose-dependent manner. Strong, dose-dependent associations were observed between fatness and soft drink consumption (positive relationship), breakfast consumption (inverse relationship) and after-school physical activity (inverse relationship). Breakfast consumption-fatness associations varied in size across ethnic groups. Lifestyle risk factors for obesity were associated with percentage differences in body composition variables that were greatest for TFM and smallest for BMI.

**Conclusions:**

Lifestyle factors were most strongly related to TFM, which suggests that studies that use BMI alone to quantify fatness underestimate the full effect of lifestyle on adiposity. This study clarifies the size of lifestyle-fatness relationships observed in previous studies.

## Background

Obesity is a major public health problem [1]. There is a strong rationale for prevention programs targeted at youth because obesity in young people tends to persist into adulthood and adolescence is a key period where lifelong behaviours form [1]. Knowledge of lifestyle determinants of obesity is important as lifestyle factors are modifiable and their identification will help to define areas that are suitable for obesity interventions in these populations.

Previous studies and systematic reviews have demonstrated that TV watching, soft drink consumption, breakfast consumption and physical inactivity are risk factors for obesity in children and adolescents [2-7]. However, past studies did not adjust for dieting intention; so, for example, sugary drink-fatness associations may be influenced by the possibility that overweight individuals are limiting their intake of sugary drinks as a way of controlling their weight. For measurement of fatness, most studies have relied on only body mass index (BMI), which has a limited ability to quantify adiposity [8]. Another drawback is that the participants in several studies had a narrow variation in level of fatness and/or exposure to lifestyle obesity risk factors. In view of these limitations, it is not clear what the “true” sizes of the relationships between the abovementioned lifestyle factors and adiposity are. Establishing the magnitude of these associations is important as it determines the level of importance of lifestyle factors in obesity aetiology. Therefore, the aim of the current study was to examine in youth who were not actively trying to lose or gain weight the associations between lifestyle variables and fatness-related body composition variables at the individual level. Our dataset allows us to clarify the size of these relationships by addressing important limitations (particularly those mentioned above) of previous studies.

## Methods

### Participants

The current study is an analysis of the baseline data collected in the Obesity Prevention In Communities (OPIC) study, an obesity intervention study with follow-up that compared changes in fatness between participating intervention and comparison sites in New Zealand, Australia, Fiji and Tonga. The participating sites were: in New Zealand, 7 schools in South Auckland with a high percentage of Pacific Island students; in Australia, 12 schools in East Geelong or the Barwon-South Western region of Victoria; in Fiji, 18 schools in Viti Levu; and, in Tonga, 4 districts in Tongatapu and Vava’u. The overall response rate (based on the number of students on the school roll) was 61% (varying from 49% to 74% by country) and a total of 17185 participated [9]. The sampling method of the OPIC study is described in more detail elsewhere [10]. All baseline data were collected between 2005 and 2006.

Ethics approval was obtained from the University of Auckland Human Participants Ethics Committee (in New Zealand), the Deakin University Human Research Ethics Committee (in Australia), National Health Research Council (NHRC) (in Fiji), the Fiji National Research Ethics Review Committee (FNRERC) Ethics Committee (in Fiji) and the Tonga National Health Ethics Research Committee (TNHERC) (in Tonga). All participants gave informed consent.

### Measurements

All measurements were carried out by trained staff using a standardised protocol. Height (±0.1 cm) was measured with a stadiometer at maximum inspiration. Impedance (±1 Ω) and body weight (±0.1 kg) were measured in light clothing (school uniform) and no socks or stockings on a Tanita BC-418 BIA device (Tanita Corp., Tokyo, Japan). BMI was calculated as body weight (kg)/height (m)^2^. This was selected as a measure of fatness as it is widely used in studies of children and adolescents. BMI z-score (BMIz) was derived from World Health Organisation Growth Standards [11]. Total fat mass (TFM) and percent body fat (%BF) were calculated using equations developed in Pacific Island, Maori, Asian and European adolescents [12].

Demographic and lifestyle data were collected via questionnaires administered through hand-held computers (personal digital assistants; PDAs) and via paper. Ethnicity was defined by self-identification. TV watching, soft drink consumption, breakfast consumption and after-school physical activity were selected for inclusion in analyses because studies and systematic reviews show that they are obesity risk factors [2-7,13], successful intervention studies exist [6,14,15], biologically plausible mechanisms can account for causal associations with fatness [2,3,5,13] and there was a large variation in exposure to these lifestyle factors (Table 1). *TV watching* was assessed by four questions: “In the last 5 school days, how many days did you watch TV, videos or DVDs (in your free time)?”, “On the last school day that you watched TV, videos or DVDs, how long did you watch for?”, “Last Saturday, how many hours did you spend watching TV, videos or DVDs?” and “Last Sunday, how many hours did you spend watching TV, videos or DVDs?” *Average daily TV viewing* was calculated as ((number of days of watching TV/videos/DVDs out of past 5 school days times the number of hours watched on the last school day) + (total number of hours spent watching TV, videos or DVDs last Saturday and Sunday combined))/7 days. The resulting values (hours per day) were categorised into three groups of approximate tertiles.

**Table 1:** Characteristics of participants

		**New Zealand**				**Fiji**		**Tonga**	**Australia**
		**Pacific**	**Maori**	**Asian**	**European**	**Indigenous Fijian**	**Fijian Indian**	**Tongan**	**Australian**
N		830	370	138	239	612	833	1019	1673
Sex	Male	405 (48.8)	161 (43.5)	64 (46.4)	119 (49.8)	271 (44.3)	388 (46.6)	471 (46.2)	1006 (60.1)
	Female	425 (51.2)	209 (56.5)	74 (53.6)	120 (50.2)	341 (55.7)	445 (53.4)	548 (53.8)	667 (39.9)
TV watching	<1 hour/day	252 (30.4)	97 (26.2)	26 (18.8)	88 (36.8)	224 (37.3)	205 (26.3)	579 (56.8)	698 (41.7)
	1-2 hours/day	241 (29.0)	120 (32.4)	51 (37.0)	66 (27.6)	196 (32.7)	272 (34.8)	268 (26.3)	562 (33.6)
	>2 hours/day	337 (40.6)	153 (41.4)	61 (44.2)	85 (35.6)	180 (30.0)	304 (38.9)	172 (16.9)	413 (24.7)
Soft drink consumption	0 cans/day	119 (15.5)	73 (21.0)	39 (29.1)	98 (42.1)	154 (25.2)	173 (20.8)	325 (31.9)	904 (54.0)
	>0-2 cans/day	508 (66.0)	216 (62.1)	79 (59.0)	117 (50.2)	373 (61.1)	537 (64.5)	562 (55.2)	703 (42.0)
	>2 cans/day	143 (18.6)	59 (17.0)	16 (11.9)	18 (7.7)	84 (13.8)	122 (14.7)	132 (13.0)	66 (4.0)
Breakfast consumption	0-2 days	271 (36.1)	128 (38.1)	37 (30.3)	54 (24.6)	129 (21.1)	117 (14.1)	163 (16.0)	190 (11.4)
	3-4 days	248 (33.1)	101 (30.1)	22 (18.0)	49 (22.3)	113 (18.5)	115 (13.9)	337 (33.1)	240 (14.4)
	5 days	231 (30.8)	107 (31.9)	63 (51.6)	117 (53.2)	370 (60.5)	597 (72.0)	519 (50.9)	1243 (74.3)
After-school physical activity	0-1 days	199 (24.0)	101 (27.3)	63 (45.7)	97 (40.6)	165 (27.5)	222 (28.1)	362 (35.5)	361 (21.6)
	2-3 days	294 (35.4)	141 (38.1)	47 (34.1)	77 (32.2)	202 (33.7)	264 (33.4)	329 (32.3)	747 (44.7)
	4-5 days	337 (40.6)	128 (34.6)	28 (20.3)	65 (27.2)	233 (38.8)	305 (38.6)	328 (32.2)	565 (33.8)
Age (years)		15.0 ± 1.5	14.8 ± 1.4	15.3 ± 1.6	15.4 ± 1.5	15.5 ± 1.3	15.4 ± 1.2	15.1 ± 2.0	14.6 ± 1.4
BMI (kg/m^2^)		25.1 ± 4.8	24.0 ± 4.9	20.4 ± 2.9	21.6 ± 3.5	21.7 ± 2.6	18.9 ± 2.8	22.4 ± 3.3	20.7 ± 2.8
BMIz		1.37 ± 0.94	1.08 ± 1.06	0.00 ± 1.00	0.37 ± 0.94	0.40 ± 0.72	-0.67 ± 1.06	0.71 ± 0.77	0.30 ± 0.86
%BF		30.4 ± 10.9	29.6 ± 11.9	21.7 ± 9.6	24.7 ± 10.6	20.4 ± 8.7	22.4 ± 8.5	22.5 ± 9.6	26.2 ± 7.9
TFM (kg)		22.9 ± 13.0	21.5 ± 13.6	12.2 ± 7.1	15.7 ± 9.6	12.3 ± 6.3	11.2 ± 5.7	14.1 ± 7.7	15.0 ± 6.2

BMI = Body mass index; BMIz = BMI z-score; %BF = Percent body fat; TFM = Total fat mass; Values are sample size (column %) or mean ± standard deviation.

Soft drink consumption was assessed by the questions, “In the last 5 school days (including time spent at home), on how many days did you have regular (non-diet) soft drinks?” and “On the last school day, how many glasses or cans of soft drinks did you have?” For the latter, each glass and can corresponded to 150 and 300 mL of soft drink, respectively, and the number of cans and corresponding number of glasses were both listed in responses to select from (for example, “1 small glass/half a can (150 mL)” and “2 small glasses/1 can (300 mL)”). *Average daily soft drink consumption* (cans/day) was calculated as (number of days of soft drink consumption times consumption on the previous day)/5 days. The amounts (cans per day) were categorised into three groups of approximate tertiles.

*Frequency of breakfast consumption* was assessed with the question, “In the last 5 school days, on how many days did you have something to eat for breakfast before school started?” *After-school physical activity* was assessed by the question, “In the last 5 school days, on how many days after school, did you do sports, dance, cultural performances or play games in which you were active?” As this may measure training/practice sessions, it may also measure physical activity (sports matches) that occurs on weekends. For both questions, responses were categorised into three groups of approximate tertiles.

Dieting and attempts to lose weight make it difficult to establish from cross-sectional data the sizes of associations between lifestyle factors and fatness [16]. Weight-loss practice can create: 1) reverse causation (mentioned in *Introduction*) and, 2) measurement error because the changing lifestyle habits no longer represent the typical lifestyle habits that contributed to the current weight, particularly if the changes occurred recently. For instance, if an individual consumed large quantities of soft drinks every day for many years, which contributed to weight gain, but consumption suddenly became low as a result of a recent attempt to reduce weight, the low consumption level would no longer represent the previous, long-term pattern of high consumption, thus making it difficult to establish the contribution of consumption to weight gain in the past. This measurement error is systematic because overweight and obese students were more likely to be trying to lose weight than normal-weight students [16]. Restricting analysis to those who were not trying to change weight circumvents these problems: through exclusion of weight-change attempts, it reduces the possibility and influence of reverse causation and reduces systematic measurement error, and this increases internal validity [17]. Therefore, *weight-change attempt* was assessed by asking each participant what he/she was doing about their weight. Students answered, “trying to lose weight”, “trying to gain weight”, “trying to stay at my current weight” or “not doing anything about my weight”. For the analyses, the first two and last two categories were combined into “change weight” and “not change weight” categories, respectively.

To provide some adjustment for possible confounding by socio-economic status (SES), Socio-economic Indexes for Areas (SEIFA) scores [18] (based on data collected from the 2001 Australian census) were measured in Australia. No measure of SES was available in the datasets for the remaining three countries. To adjust for potential confounding arising from the possibility that overweight/obese individuals may be less likely to participate in sports because of fears of being teased [7], students were asked to rate how often other teenagers teased them (responses ranging from “never” to “almost always”).

### Statistical analysis

In order to correct standard errors for design effects from clustered sampling, SUDAAN (version 10.0) was used for all analyses. Statistical significance was set at P < 0.05. All continuous variables were examined for normality.

Associations between lifestyle and body composition variables were examined by multiple linear regression. All models were adjusted for sex and age. Models for country-specific analyses and for all ethnic groups combined were additionally adjusted for ethnicity. Attempts to change weight have been shown to moderate relationships between lifestyle variables and fatness [16]. To examine whether this was the case in the current study, an interaction term consisting of the product of lifestyle and weight-change attempt (binary variable comprising the abovementioned “change weight” and “not change weight” categories) was added to models, adjusted for sex, age, ethnicity, lifestyle factor and weight-change attempt. If the interaction was found to be significant, all subsequent analyses were restricted to those who were in the “not change weight” category. The entire OPIC questionnaire (questions are listed elsewhere [9]) was reviewed to identify potential confounders by examining correlations with fatness and the four lifestyle factors (TV watching, soft drink consumption, breakfast consumption and after-school physical activity). Some variables, such as snack food consumption [9], were considered to be mediators [2,3,5,13] in causal lifestyle-fatness pathways and it may not be appropriate to adjust for these. Following this, identified potential confounders (SEIFA and teasing, described in *Measurements* section) were initially added to models, but as their inclusion did not alter effect sizes by at least 10%, they were removed. The Wald F-test was used to assess whether associations were dose-dependent (that is, whether there were stepwise differences in fatness outcomes with stepped differences in exposure).

Using Review Manager version 5.0 (Nordic Cochrane Centre, Copenhagen), forest plots were constructed to illustrate the associations between the lifestyle and body composition variables. These showed the effect sizes and associated 95% confidence intervals for each ethnic group. Overall estimates of the pooled relation were calculated using inverse-variance weighting and with the use of random-effects models.

## Results

Sample sizes in each of the lifestyle exposure groups, by weight-control attempt, are provided in Additional file 1. Lifestyle-fatness associations (all ethnic groups combined; adjusted for age, sex and ethnicity) among those not trying to change weight differed from corresponding ones in the “change weight” group with respect to strength and/or direction. That is, TV watching and soft drink consumption relationships were negative (positive for the “not change weight” group, as detailed below), breakfast consumption associations were stronger and after-school physical activity relationships were weaker (Additional file 2). This is reflected in the fact that weight-change attempt moderated associations between all lifestyle and body composition variables (P-values for interactions ranging from 0.054 to <0.0001). Therefore, those who were in the “change weight” category were excluded from all further analyses. Characteristics of the remaining participants – those who were not trying to gain or lose weight (n = 5714) – are provided in Table 1. The sample comprised 8 ethnic groups: Pacific Island, Maori, Asian and European (all four from New Zealand), Australian (from Australia), Tongan (from Tonga) and Indigenous Fijian and Fijian Indian (both from Fiji). Age ranged from 12 to 22 years. Not all participants answered all lifestyle questions so that total sample sizes for each of the lifestyle factors varied slightly.

### Dose-related associations between lifestyle and body composition

Dose-related associations of lifestyle variables with body composition variables – by ethnic group and country, and among all ethnic groups combined – are shown in Tables 2, 3, 4 and 5. For TV watching (Table 2), among all ethnic groups combined, BMI, %BF and TFM were higher among those who watched TV for >2 hours per day than among those who watched for <1 hour per day (P = 0.018, 0.0019 and 0.0007, respectively). In addition, there were dose-related effects of increasing BMI, %BF and TFM with increasing TV exposure (P = 0.047, 0.0077 and 0.0031, respectively). Table 3 shows that, among all ethnic groups combined, soft drink consumption had positive and dose-dependent associations with body composition variables (P = 0.0022, 0.0029, 0.035 and 0.0091, for BMI, BMIz, %BF and TFM, respectively). For breakfast consumption (Table 4), there were inverse, dose-dependent associations between breakfast consumption and body composition variables among all ethnic groups combined (P = 0.0001, 0.0004, 0.0019 and 0.0024, for BMI, BMIz, %BF and TFM, respectively). Table 5 shows that, among all ethnic groups combined, the relationships between after-school physical activity and body composition variables were inverse and dose-dependent (P = 0.0010, 0.020, <0.0001 and <0.0001, for BMI, BMIz, %BF and TFM respectively).

**Table 2:** **Relationship between TV watching and body composition variables**^**a**^

		**New Zealand**					**Fiji**			**Tonga**	**Australia**	**All**
	**Hours per day**	**Pacific (N = 830)**	**Maori (N = 370)**	**Asian (N = 138)**	**European (N = 239)**	**All New Zealand (N = 1577)**	**Indigenous Fijian (N = 600)**	**Fijian Indian (N = 781)**	**All Fiji (N = 1381)**	**Tongan (N = 1019)**	**Australian (N = 1673)**	**All (N = 5650)**^**d**^
Mean BMI^b^	<1	24.99 (0.17)	23.96 (0.50)	19.68 (0.43)	21.04 (0.39)	23.68 (0.24)	21.68 (0.23)	18.94 (0.20)	20.13 (0.16)	22.26 (0.15)	20.62 (0.07)	21.64 (0.08)
Increment in BMI^c^	1-2	0.06 (0.45)	0.23 (0.52)	1.21 (0.42)^†^	0.52 (0.13)^‡^	0.31 (0.26)	-0.13 (0.28)	-0.12 (0.18)	-0.13 (0.18)	0.23 (0.19)	0.21 (0.14)	0.16 (0.09)
	>2	0.33 (0.29)	-0.00 (0.51)	0.69 (0.34)*	1.18 (0.29)^‡^	0.41 (0.26)	-0.00 (0.29)	-0.12 (0.21)	-0.08 (0.18)	0.43 (0.21)*	0.15 (0.10)	0.23 (0.09)*
												
Mean BMIz^b^	<1	1.35 (0.04)	1.09 (0.11)	-0.26 (0.15)	0.21 (0.10)	0.97 (0.06)	0.42 (0.07)	-0.63 (0.08)	-0.18 (0.05)	0.68 (0.04)	0.28 (0.03)	0.43 (0.02)
Increment in BMIz^c^	1-2	0.01 (0.01)	0.02 (0.10)	0.39 (0.12)^†^	0.16 (0.06)^†^	0.07 (0.06)	-0.04 (0.08)	-0.07 (0.07)	-0.06 (0.06)	0.06 (0.05)	0.04 (0.04)	0.03 (0.03)
	>2	0.06 (0.06)	-0.03 (0.12)	0.27 (0.11)*	0.31 (0.08)^‡^	0.09 (0.06)	-0.02 (0.08)	-0.06 (0.08)	-0.04 (0.06)	0.09 (0.05)	0.02 (0.03)	0.05 (0.03)
												
Mean %BF^b^	<1	29.82 (0.52)	28.92 (1.44)	18.89 (1.35)	23.09 (1.15)	27.63 (0.52)	20.50 (0.62)	22.63 (0.60)	21.71 (0.43)	22.08 (0.60)	26.07 (0.36)	24.67 (0.23)
Increment in %BF^c^	1-2	0.88 (1.22)	1.55 (1.47)	3.85 (2.51)	1.48 (1.68)	1.46 (0.77)	-0.40 (0.70)	-0.45 (0.65)	-0.45 (0.54)	0.54 (0.64)	0.11 (0.44)	0.43 (0.33)
	>2	0.80 (0.73)	0.37 (1.59)	3.20 (1.32)*	3.43 (0.88)^‡^	1.28 (0.46)*	0.19 (0.67)	-0.63 (0.56)	-0.30 (0.44)	1.61 (0.53)^†^	0.37 (0.38)	0.77 (0.24)^†^
												
Mean TFM^b^	<1	22.35 (0.67)	20.95 (1.61)	9.98 (0.97)	14.26 (1.03)	19.69 (0.60)	12.27 (0.47)	11.27 (0.45)	11.72 (0.33)	13.69 (0.46)	14.76 (0.22)	15.19 (0.20)
Increment in TFM^c^	1-2	0.64 (1.66)	1.33 (1.85)	3.33 (1.79)	1.25 (0.68)	1.21 (0.84)	-0.19 (0.58)	-0.20 (0.43)	-0.25 (0.43)	0.50 (0.48)	0.49 (0.36)	0.50 (0.28)
	>2	0.98 (1.09)	0.36 (1.63)	2.28 (0.94)*	3.15 (0.83)^‡^	1.27 (0.54)	0.32 (0.61)	-0.31 (0.43)	-0.08 (0.34)	1.44 (0.55)*	0.47 (0.28)	0.75 (0.21)^‡^

^a^Adjusted for age and sex. “New Zealand All”, “Fiji All” and “All countries” were further adjusted for ethnicity; ^b^Standard error in parentheses; ^c^Compared to “<1 hour per day” (reference category). Standard error in parentheses; ^d^R^2^ for BMI, BMIz, %BF and TFM models = 0.315, 0.311, 0.243 and 0.227, respectively; BMI = Body mass index (kg/m^2^); BMIz = BMI z-score; %BF = Percent body fat (%); TFM = Total fat mass (kg); *P<0.05; ^†^P < 0.01; ^‡^P < 0.001.

**Table 3:** **Relationship between average daily soft drink consumption and body composition variables**^**a**^

		**New Zealand**					**Fiji**			**Tonga**	**Australia**	**All**
	**Cans per day**	**Pacific (N = 770)**	**Maori (N = 348)**	**Asian (N = 134)**	**European (N = 233)**	**All New Zealand (N = 1485)**	**Indigenous Fijian (N = 611)**	**Fijian Indian (N = 832)**	**All Fiji (N = 1443)**	**Tongan (N = 1019)**	**Australian (N = 1673)**	**All (N = 5620)**^**d**^
Mean BMI^b^	0	25.10 (0.40)	23.76 (0.63)	20.52 (0.40)	21.21 (0.27)	23.70 (0.35)	21.69 (0.26)	18.87 (0.20)	20.05 (0.15)	21.95 (0.14)	20.68 (0.10)	21.52 (0.10)
Increment in BMI^c^	>0-2	0.04 (0.46)	-0.13 (0.61)	-0.33 (0.33)	0.56 (0.32)	0.11 (0.34)	-0.17 (0.23)	0.09 (0.22)	-0.01 (0.13)	0.46 (0.14)^†^	0.07 (0.14)	0.17 (0.10)
	>2	0.12 (0.60)	2.03 (0.86)*	0.73 (0.63)	1.79 (0.69)*	0.88 (0.52)	0.52 (0.44)	-0.14 (0.38)	0.16 (0.29)	1.42 (0.25)^‡^	0.46 (0.23)*	0.75 (0.21)^‡^
												
Mean BMIz^b^	0	1.36 (0.08)	1.00 (0.15)	0.05 (0.12)	0.26 (0.07)	0.97 (0.08)	0.40 (0.08)	-0.67 (0.08)	-0.22 (0.06)	0.60 (0.04)	0.28 (0.03)	0.38 (0.03)
Increment in BMIz^c^	>0-2	0.01 (0.09)	0.00 (0.14)	-0.14 (0.09)	0.13 (0.07)	0.03 (0.08)	-0.04 (0.07)	0.01 (0.09)	-0.01 (0.05)	0.11 (0.04)*	0.02 (0.04)	0.05 (0.03)
	>2	0.04 (0.12)	0.51 (0.20)*	0.27 (0.17)	0.53 (0.17)^†^	0.23 (0.12)	0.17 (0.13)	-0.05 (0.16)	0.04 (0.11)	0.37 (0.07)^‡^	0.15 (0.07)*	0.20 (0.06)^‡^
												
Mean %BF^b^	0	31.10 (1.07)	28.94 (1.45)	21.96 (0.54)	24.22 (0.53)	28.48 (0.82)	20.78 (0.64)	22.29 (0.51)	21.61 (0.41)	21.16 (0.58)	26.30 (0.26)	24.59 (0.28)
Increment in %BF^c^	>0-2	-0.88 (1.12)	-0.43 (1.28)	-0.59 (0.89)	0.27 (0.94)	-0.36 (0.85)	-0.70 (0.45)	0.23 (0.61)	-0.11 (0.38)	1.64 (0.47)^‡^	-0.22 (0.44)	0.36 (0.31)
	>2	-0.81 (1.52)	5.38 (1.93)^†^	1.94 (1.23)	6.01 (3.38)	1.84 (1.48)	0.77 (1.04)	-0.50 (0.81)	0.13 (0.70)	3.32 (0.43)^‡^	-0.17 (0.70)	1.49 (0.56)*
												
Mean TFM^b^	0	22.91 (1.27)	20.67 (1.75)	12.34 (0.54)	15.11 (0.44)	20.07 (0.97)	12.53 (0.54)	11.19 (0.41)	11.69 (0.34)	12.89 (0.42)	15.05 (0.20)	15.03 (0.25)
Increment in TFM^c^	>0-2	-0.04 (1.34)	-0.41 (1.69)	-0.49 (0.51)	0.56 (1.03)	0.06 (1.02)	-0.57 (0.44)	0.21 (0.47)	-0.04 (0.33)	1.38 (0.36)^‡^	-0.02 (0.31)	0.40 (0.28)
	>2	0.32 (1.68)	6.23 (2.45)*	1.66 (0.94)	5.70 (2.83)*	2.58 (1.54)	1.11 (0.96)	-0.58 (0.70)	0.28 (0.63)	3.16 (0.45)^‡^	0.09 (0.64)	1.76 (0.55)^†^

^a^Adjusted for age and sex. “New Zealand All”, “Fiji All” and “All countries” were further adjusted for ethnicity; ^b^Standard error in parentheses; ^c^Compared to “0 cans per day” (reference category). Standard error in parentheses; ^d^R^2^ for BMI, BMIz, %BF and TFM models = 0.312, 0.310, 0.245 and 0.225, respectively; BMI = Body mass index (kg/m^2^); BMIz = BMI z-score; %BF = Percent body fat (%); TFM = Total fat mass (kg); *P<0.05; ^†^P <0.01; ^‡^P <0.001.

**Table 4:** **Relationship between frequency of breakfast consumption and body composition variables**^**a**^

		**New Zealand**					**Fiji**			**Tonga**	**Australia**	**All**
	**Days**	**Pacific (N = 750)**	**Maori (N = 336)**	**Asian (N = 122)**	**European (N = 220)**	**All New Zealand (N = 1428)**	**Indigenous Fijian (N = 612)**	**Fijian Indian (N = 829)**	**All Fiji (N = 1441)**	**Tongan (N = 1019)**	**Australian (N = 1673)**	**All (N = 5561)**^**d**^
Mean BMI^b^	5	24.17 (0.18)	22.74 (0.33)	20.09 (0.24)	21.29 (0.28)	23.10 (0.08)	21.51 (0.13)	18.77 (0.12)	19.94 (0.09)	22.31 (0.12)	20.65 (0.07)	21.47 (0.06)
Increment in BMI^c^	3-4	1.22 (0.34)^‡^	2.10 (0.87)*	0.25 (0.16)	0.09 (0.16)	1.16 (0.30)^†^	0.41 (0.32)	0.39 (0.35)	0.36 (0.23)	0.22 (0.18)	0.12 (0.30)	0.43 (0.15)^†^
	0-2	1.60 (0.35)^‡^	1.88 (0.57)^†^	0.94 (0.20)^‡^	0.75 (0.61)	1.43 (0.20)^‡^	0.34 (0.25)	0.53 (0.25)*	0.42 (0.20)	0.09 (0.24)	0.57 (0.18)^†^	0.64 (0.14)^‡^
												
Mean BMIz^b^	5	1.18 (0.04)	0.82 (0.07)	-0.09 (0.09)	0.29 (0.06)	0.86 (0.02)	0.36 (0.04)	-0.71 (0.05)	-0.26 (0.03)	0.69 (0.04)	0.28 (0.03)	0.38 (0.02)
Increment in BMIz^c^	3-4	0.24 (0.07)^‡^	0.43 (0.19)*	0.01 (0.05)	-0.01 (0.05)	0.23 (0.06)*	0.09 (0.08)	0.12 (0.13)	0.10 (0.06)	0.06 (0.05)	0.01 (0.01)	0.09 (0.04)*
	0-2	0.30 (0.07)^‡^	0.40 (0.12)^†^	0.29 (0.06)^‡^	0.19 (0.15)	0.29 (0.04)^‡^	0.08 (0.07)	0.18 (0.09)*	0.13 (0.07)	0.04 (0.07)	0.14 (0.05)^†^	0.14 (0.03)^‡^
												
Mean %BF^b^	5	28.81 (0.72)	26.81 (0.74)	22.31 (0.75)	23.81 (0.47)	27.14 (0.49)	20.30 (0.42)	22.09 (0.41)	21.34 (0.34)	22.21 (0.58)	25.93 (0.23)	24.49 (0.17)
Increment in %BF^c^	3-4	2.18 (1.14)	4.54 (1.76)*	-2.14 (1.25)	-0.44 (0.86)	2.04 (0.84)	0.74 (0.56)	1.07 (0.83)	0.78 (0.47)	0.68 (0.42)	0.30 (0.77)	0.95 (0.33)^†^
	0-2	2.55 (0.99)*	3.99 (1.35)^†^	0.18 (0.98)	2.89 (1.31)*	2.58 (0.83)*	0.06 (0.42)	0.77 (0.54)	0.41 (0.40)	0.41 (0.66)	1.96 (0.51)^‡^	1.21 (0.34)^‡^
												
Mean TFM^b^	5	20.54 (0.84)	18.21 (0.76)	12.17 (0.48)	14.81 (0.56)	18.55 (0.48)	12.14 (0.30)	10.99 (0.31)	11.50 (0.26)	13.86 (0.41)	14.86 (0.15)	14.93 (0.16)
Increment in TFM^c^	3-4	3.31 (1.27)*	5.09 (2.28)*	-0.76 (0.59)	-0.14 (0.63)	2.91 (0.88)*	0.92 (0.64)	0.78 (0.75)	0.69 (0.49)	0.54 (0.35)	0.25 (0.67)	1.02 (0.35)^†^
	0-2	3.60 (1.18)^†^	4.78 (1.43)^†^	0.94 (0.51)	2.98 (1.46)*	3.37 (0.81)^†^	0.13 (0.46)	0.89 (0.54)	0.51 (0.40)	0.17 (0.51)	1.31 (0.44)^†^	1.33 (0.37)^‡^

^a^Adjusted for age and sex. “New Zealand All”, “Fiji All” and “All countries” were further adjusted for ethnicity; ^b^Standard error in parentheses; ^c^Compared to “5 days” (reference category). Standard error in parentheses; ^d^R^2^ for BMI, BMIz, %BF and TFM models = 0.317, 0.311, 0.250 and 0.230, respectively; BMI = Body mass index (kg/m^2^); BMIz = BMI z-score; %BF = Percent body fat (%); TFM = Total fat mass (kg); *P<0.05; ^†^P <0.01; ^‡^P <0.001.

**Table 5:** **Relationship between after-school physical activity and body composition variables**^**a**^

		**New Zealand**					**Fiji**			**Tonga**	**Australia**	**All**
	**Days**	**Pacific (N = 830)**	**Maori (N = 370)**	**Asian (N = 138)**	**European (N = 239)**	**All New Zealand (N = 1577)**	**Indigenous Fijian (N = 600)**	**Fijian Indian (N = 791)**	**All Fiji (N = 1391)**	**Tongan (N = 1019)**	**Australian (N = 1673)**	**All (N = 5660)**^**d**^
Mean BMI^b^	4-5	24.45 (0.20)	23.95 (0.21)	20.29 (0.43)	21.34 (0.33)	23.47 (0.15)	21.47 (0.11)	18.83 (0.15)	19.98 (0.10)	22.17 (0.14)	20.66 (0.10)	21.54 (0.06)
Increment in BMI^c^	2-3	0.89 (0.18)^‡^	-0.24 (0.23)	0.13 (0.37)	0.41 (0.41)	0.50 (0.18)*	0.03 (0.16)	0.09 (0.26)	0.05 (0.14)	0.16 (0.16)	0.16 (0.14)	0.23 (0.08)^†^
	0-1	1.58 (0.15)^‡^	0.63 (0.39)	0.22 (0.42)	0.33 (0.20)	0.97 (0.23)^†^	0.57 (0.25)*	-0.02 (0.21)	0.21 (0.18)	0.49 (0.17)^†^	-0.01 (0.09)	0.46 (0.12)^‡^
												
Mean BMIz^b^	4-5	1.24 (0.04)	1.08 (0.04)	0.03 (0.14)	0.29 (0.04)	0.95 (0.03)	0.36 (0.04)	-0.69 (0.05)	-0.23 (0.04)	0.67 (0.04)	0.29 (0.03)	0.41 (0.02)
Increment in BMIz^c^	2-3	0.16 (0.04)^‡^	-0.06 (0.08)	-0.05 (0.12)	0.12 (0.14)	0.09 (0.04)	-0.01 (0.06)	0.03 (0.10)	0.01 (0.06)	0.01 (0.05)	0.03 (0.04)	0.04 (0.02)
	0-1	0.30 (0.04)^‡^	0.08 (0.08)	-0.02 (0.13)	0.08 (0.08)	0.17 (0.05)*	0.13 (0.07)	-0.02 (0.07)	0.04 (0.06)	0.11 (0.05)*	-0.03 (0.03)	0.08 (0.03)^†^
												
Mean %BF^b^	4-5	29.06 (0.32)	29.67 (0.52)	20.63 (1.50)	23.09 (1.11)	27.62 (0.28)	19.60 (0.39)	22.06 (0.36)	21.04 (0.30)	21.36 (0.64)	25.55 (0.43)	24.15 (0.19)
Increment in %BF^c^	2-3	1.16 (0.42)^†^	-0.27 (0.50)	0.93 (1.50)	3.03 (0.94)^†^	0.99 (0.31)*	0.77 (0.47)	0.38 (0.79)	0.50 (0.45)	0.89 (0.46)	0.81 (0.43)	0.92 (0.21)^‡^
	0-1	3.88 (0.56)^‡^	0.02 (1.03)	1.72 (0.82)*	1.61 (0.50)^†^	2.12 (0.63)*	2.06 (0.60)^†^	0.25 (0.58)	0.94 (0.41)*	2.39 (0.50)^‡^	1.32 (0.47)^†^	1.96 (0.29)^‡^
												
Mean TFM^b^	4-5	21.27 (0.50)	21.44 (0.62)	11.36 (1.30)	14.36 (1.20)	19.42 (0.35)	11.68 (0.31)	10.94 (0.31)	11.30 (0.25)	13.23 (0.44)	14.59 (0.28)	14.82 (0.16)
Increment in TFM^c^	2-3	1.69 (0.53)^†^	-0.45 (0.38)	0.80 (1.17)	2.64 (0.82)^†^	1.18 (0.35)*	0.51 (0.37)	0.33 (0.55)	0.36 (0.31)	0.66 (0.37)	0.66 (0.36)	0.78 (0.18)^‡^
	0-1	4.42 (0.57)^‡^	0.97 (1.31)	1.29 (0.76)	1.28 (0.63)*	2.53 (0.64)^†^	1.62 (0.53)^†^	0.20 (0.43)	0.71 (0.36)	1.76 (0.42)^‡^	0.76 (0.26)^†^	1.64 (0.29)^‡^

^a^Adjusted for age and sex. “New Zealand All”, “Fiji All” and “All countries” were further adjusted for ethnicity; ^b^Standard error in parentheses; ^c^Compared to “4-5 days” (reference category). Standard error in parentheses; ^d^R^2^ for BMI, BMIz, %BF and TFM models = 0.317, 0.313, 0.247 and 0.230, respectively; BMI = Body mass index (kg/m^2^); BMIz = BMI z-score; %BF = Percent body fat (%); TFM = Total fat mass (kg); *P<0.05; ^†^P <0.01; ^‡^P <0.001.

### Consistency of associations across ethnic groups

**Figure 1:**
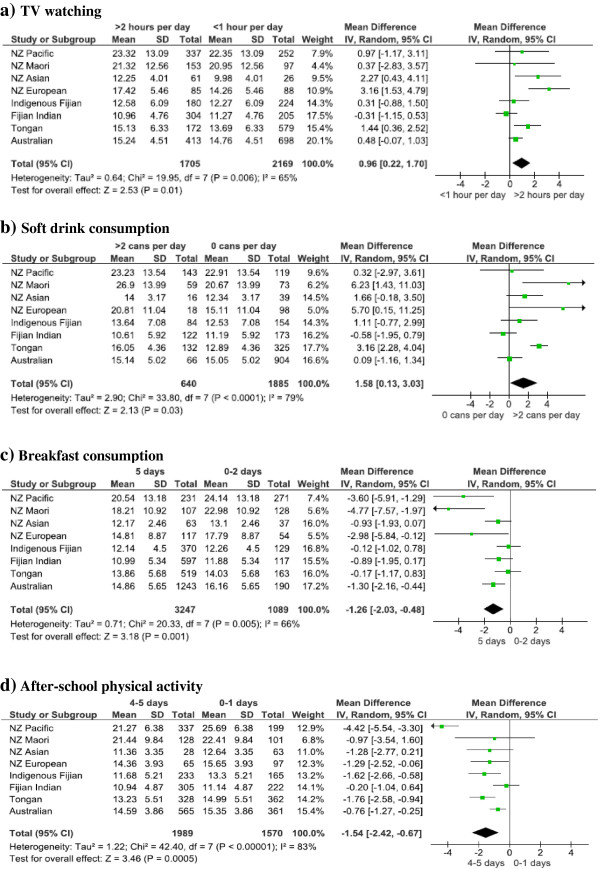
**Forest plots of relationships between lifestyle variables and total fat mass (adjusted for age and sex). a)** TV watching (<1 hour/day versus >2 hours/day), **b)** Soft drink consumption (0 cans/day versus >2 cans/day), **c)** Breakfast consumption (5 days versus 0, 1 or 2 days), **d)** After-school physical activity (4–5 days versus 0–1 days). NZ = New Zealand.

TFM differences between highest and lowest lifestyle exposure categories tabulated in Tables 2, 3, 4 and 5 are illustrated in Figure 1. Pooled effects were positive for TV watching (P = 0.01) and soft drink consumption (P = 0.03) and inverse for breakfast consumption (P = 0.001) and after-school physical activity (P = 0.0005). The direction of each of these effects was consistent across the 8 ethnic groups: 7 out of 8 associations were positive for TV watching and soft drink consumption, while 8 out of 8 associations were inverse for breakfast consumption and after-school physical activity. Of note, effect sizes for breakfast consumption were largest among New Zealand Pacific and Maori groups.

### Variable strength of associations with different body composition variables

**Figure 2:**
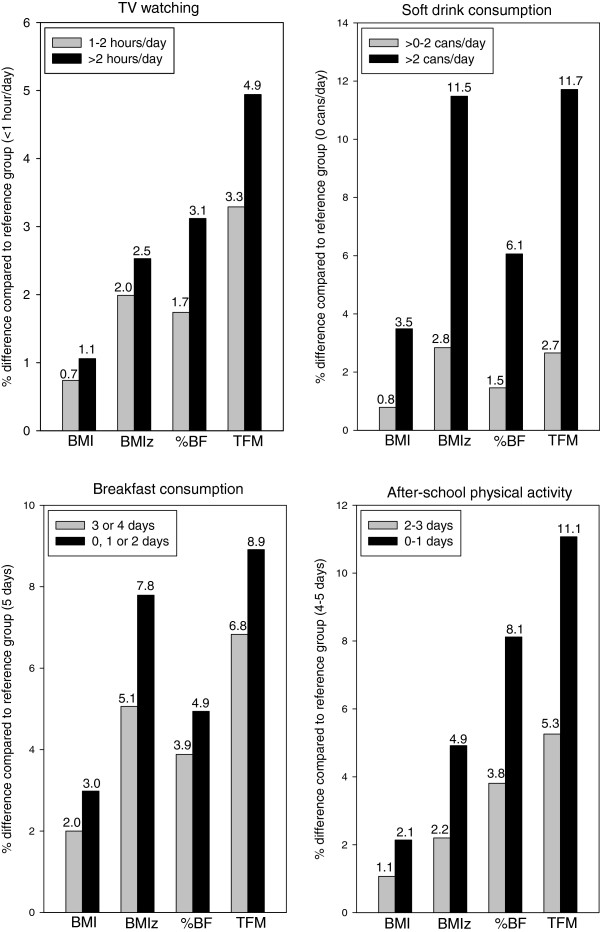
**Relationship between lifestyle and body composition variables (as a % of mean values of reference group from Tables**2**,**3**,**4**,**5**) in all ethnic groups combined.** BMI = Body mass index; BMIz = BMI z-score (values are derived from transforming mean BMIz values from regression analyses into percentiles); %BF = Percent body fat; TFM = Total fat mass.

To compare the strength of lifestyle-fatness associations across body composition variables, overall (all ethnic groups combined) differences in BMI, %BF and TFM tabulated in Tables 2, 3, 4 and 5 were expressed as a percentage of mean values in the corresponding reference group category (tabulated) and the resulting values were graphed in Figure 2. This was done for BMIz too, but the differences and reference group mean values used in the calculations were percentiles derived by converting the mean BMIz values across the lifestyle exposure groups into percentile values. This transformation was applied in order to avoid having denominators (reference group mean values) that may be ≤0 in the calculations when raw z-score values are used. As shown in Figure 2, for all 4 lifestyle variables, differences in lifestyle exposure groups were associated with percentage differences in body composition variables that were greatest for TFM, followed by either BMIz or %BF and then BMI.

## Discussion

This study showed that TV watching was positively related to fatness in a dose-dependent manner. Strong, dose-dependent associations between fatness and soft drink consumption (positive relationship), breakfast consumption (inverse relationship) and after-school physical activity (inverse relationship) were observed. These associations were independent of ethnicity, sex and age. Along with their effect sizes, a highlight of our study was that differences in lifestyle obesity risk factors were associated with percentage differences in body composition variables that were greatest for TFM, followed by either BMIz or %BF and then BMI.

The positive TV watching- and soft drink-fatness associations and the inverse breakfast consumption- and physical activity-fatness relationships we observed have previously been reported in studies carried out internationally [2-7]. However, our study differs from those studies in that we adjusted for dieting intention and quantified %BF and TFM.

Of particular interest was the size of the associations. In the present study, compared to the low-exposure categories, BMI in the high-exposure categories was between 0.23 and 0.75 kg/m^2^ higher (Tables 2, 3, 4 and 5) or between 1.1 to 3.5% higher (Figure 2). These effect sizes are larger than those reported in meta-analyses and several previous studies [2,3,5]. This difference may reflect strengths of our study (discussed below) – in particular, the large variation in fatness level and exposure to lifestyle factors (Table 1), together with accounting for dieting intention in analyses.

Change in the level of exposure to lifestyle obesity risk factors, such as an increase in soft drink consumption, alters fat stores through change in energy intake or expenditure. BMIz, %BF and, in particular, BMI have a limited ability to reflect these changes in body composition as they are influenced by total fat-free mass. In addition, increases in TFM will be 'underestimated’ by the changes in %BF since the latter is based on the ratio of TFM and body weight, both of which increase with increasing TFM. Consistent with this, the present study showed that lifestyle variables were most strongly related to TFM and had weaker associations with BMIz, %BF and BMI. To our knowledge, this is the first study to show this in children and adolescents; the relative strength of associations of these four body composition variables with lifestyle factors has not been formally assessed by previous work.

Across ethnic groups, TV watching and soft drink consumption associations were consistently positive, while the breakfast consumption and after-school physical activity associations were consistently inverse. TV watching relationships were dose-dependent, while soft drink consumption, breakfast consumption and after-school physical activity relationships were strong plus dose-dependent. Of note, associations were especially strong for soft drink consumption and after-school physical activity, with TFM percentage differences between highest and lowest exposure categories exceeding 11% (Figure 2). In the light of successful intervention studies [6,14,15], the consistent, strong and dose-dependent nature of these associations gives evidence that they may well be causal.

In the Pacific region (defined in this paper as Australia, New Zealand and other Pacific Island countries), the prevalence of obesity is among the highest in the world [19]. Due to the geographical location of our participants, this study has the ability to provide particularly relevant evidence for youth in the Pacific region. Previous studies similar to ours have a limited ability to do so, especially since they were small, used BMI alone as a fatness measure, did not adjust for dieting intention or studied mainly primary-school aged children [16,18,20-44]. Given that our study addressed these drawbacks, it provides stronger evidence of whether lifestyle factors contribute to obesity in Pacific region youth. In addition, our study defines a type of physical activity (after-school physical activity) that is predictive of fatness, which is important because this helps to specify an appropriate physical activity intervention target. Few studies have done this [18,41]; nearly all have measured *overall* physical activity (such as daily step counts) instead.

This study has a unique ability to explain ethnic disparities in fatness levels that exist among adolescents in the Pacific region (Table 1, [9]). Of note, fatness levels are especially high among New Zealand Pacific Island and Maori youth (Table 1, [9]), indicating a need for obesity interventions targeted at these groups. These disparities are partly due to the fact that high TV watching, high soft drink consumption and breakfast skipping are more prevalent in these groups (Table 1, [9]). Breakfast skipping is a particularly important explanation as its association with fatness is strongest in these groups (Table 4 and Figure 1). This may be attributed to ethnic differences in factors that determine the level of consumption of unhealthy (energy-dense) food outside of home – a plausible mediator in the breakfast skipping-fatness association. These factors include: 1) healthiness and accessibility of school food options outside of home (in school canteens and shops) and, 2) spending money allocated for school food purchase.

This study is the largest that we are aware of carried out in children and adolescents in the Pacific region [16,18,20-44] and larger than most of those performed outside the Pacific region [2-4,7]. Other study strengths are that physical measurements were objectively measured (not self-reported), the use of validated %BF and TFM measures, the large variation in fatness level and exposure to lifestyle factors (Table 1), and the ethnically and geographically diverse nature of the sample. Furthermore, serving size was included in the assessment of soft drink consumption; this is often not the case [3]. Finally, the large number of sub-samples (8 ethnic groups) and homogenous measurements across these facilitated assessment of consistency of associations. Studies that have examined consistency have utilised fewer sub-groups or have pooled together results of different studies with heterogenous measurements.

A limitation of this study is error inherent in the measurement of the lifestyle variables. Random measurement error associated with the lifestyle variables – resulting from day-to-day variation in lifestyle habits and imperfect memory to recall these – would weaken associations. Therefore, the associations may well be stronger than we observed. Being cross-sectional, this study is unable to rule out the possibility of reverse causation. However, this possibility and any influence of reverse causation were reduced for some reasons. Firstly, analysis was restricted to those who said they were not trying to change weight, so the trigger for reverse causality (trying to lose weight) would have been minimal. Secondly, lifestyle factors were most strongly related to TFM, followed by %BF and then BMI, and this hierarchical pattern would fit a forward causation (changes in energy intake or expenditure leading to changes in TFM, as discussed above) but a reverse-causation mechanism would be unlikely to produce this hierarchy of strength of relationships. In addition, if reverse causation did account for the relationships between physical activity and fatness, fear of being teased may act as a mediator [7]. However, when a measure of this mediator was adjusted for, the physical activity-fatness associations remained significant (data not shown), which gives some evidence to suggest that reverse causation did not fully explain these associations.

The fact that analysis was largely restricted to those who were not trying to change weight would have limited the ability to extrapolate findings to those trying to change weight. Because of the improvement in internal validity it provided (as discussed in *Measurements*), given that a notable fraction of participants were trying to change weight (Additional file 1), this restriction was considered to be – by us and Rothman *et al.*[17] – important and justifiable. However, our findings have at least some applicability to the “change weight” group because individuals from this group probably would have previously made no attempts to change weight, which is supported from epidemiological evidence that weight-control attempts are less prevalent in childhood than in adolescence [45]. In other words, our results suggest that lifestyle factors may well have contributed to weight gain of individuals before they tried to change weight.

With regard to SES confounding, the participants were recruited from schools with similar SES and there was low variation in personal SES in the areas sampled from in New Zealand [16]. Further, in Australia, analyses showed that inclusion of SEIFA scores in statistical models did not alter our conclusions (data not shown). These factors reduce the possibility of confounding by SES.

We did not measure pubertal status, which may have been a covariate worth controlling for in statistical models. However, maturational stage is correlated with age and sex, and may have varied with ethnicity in our dataset [46]. Thus, at least some adjustment for puberty would have been provided through the inclusion of age, sex and ethnicity in models. Further, any correlation between pubertal stage and lifestyle factors might be mediated by weight-control attempt [47], but we accounted for the latter in the analyses.

The self-reported nature of the lifestyle data collection raises the possibility of there being social desirability bias associated with the measurement of the lifestyle variables. For instance, obese adolescents may have under-reported their intake of soft drinks because of their unhealthy connotation. However, any influence on the results from this source of error was minimised by indicating to the students that all collected data were confidential and having each student answer survey questions alone.

## Conclusions

Our findings support the view that TV watching, soft drink consumption, breakfast consumption and after-school physical activity are important determinants of fatness in youth. Therefore, this study supports the reduction in TV watching and soft drink consumption and encouragement of breakfast consumption and physical activity (particularly after school). For reducing ethnic disparities in fatness, increasing breakfast consumption is an especially appropriate strategy as breakfast skipping was most common and had the strongest relationship with fatness in New Zealand Pacific and Maori groups. Intervention studies are required to determine effective ways of carrying out these recommendations. Lifestyle factors have the strongest association with TFM and the weakest relationship with BMI, which reflects the limited usefulness of the latter as a measure of TFM. This suggests that obesity interventions and studies that use BMI alone to quantify fatness underestimate the full effect of lifestyle factors on adiposity. This is of great significance because BMI is widely used to measure body fat. For example, conclusions made by many studies on the effectiveness of obesity interventions [48] and by lifestyle-fatness meta-analyses on effect sizes [3,5,49,50] are based on the use of BMI as a fatness measure.

## Abbreviations

BMI: Body mass index; BMIz: Body mass index z-score; %BF: Percent body fat; TFM: Total fat mass; SES: Socio-economic status; OPIC: Obesity prevention in communities; SEIFA: Socio-economic indexes for areas.

## Competing interests

The authors declare that they have no competing interests.

## Authors’ contributions

JS acquired data for the OPIC study, contributed to the conception of the manuscript research questions, performed all statistical analyses and drafted the manuscript. RS contributed to study conception and design, acquired data, contributed to the conception of the manuscript research questions and the interpretation of findings, provided advice for statistical analysis and critically reviewed the manuscript. LP participated in the interpretation of findings and critically reviewed the manuscript. Both GW and KF collected data and participated in critical revision of the manuscript. BS contributed to study conception and design, contributed to the interpretation of findings and critically reviewed the manuscript. All authors read and approved the final manuscript.

## Supplementary Material

Additional file 1 **Lifestyle characteristics of all participants by weight-control attempt.** Sample numbers in lifestyle exposure categories stratified by weight-control attempt. Click here for file

Additional file 2 **Relationships between lifestyle and body composition variables (adjusted for age, sex and ethnicity) by weight-control attempt and among all participants.** Graphs of lifestyle-fatness associations. Click here for file
