# Supplementary material for: Sizing the association between lifestyle behaviours and fatness in a large, heterogeneous sample of youth of multiple ethnicities from 4 countries
Source: Int J Behav Nutr Phys Act. 2013 Oct 12;10:115. doi: 10.1186/1479-5868-10-115 (PMC3853713; doi:10.1186/1479-5868-10-115)
Supplement: Additional file 1 — Lifestyle characteristics of all participants by weight-control attempt. Sample numbers in lifestyle exposure categories stratified by weight-control attempt. [file 1479-5868-10-115-S1.pdf]

Lifestyle characteristics of all participants by weight-control attempt

Lifestyle variable	Weight-control attempt		All weight-control attempt groups combined
	Lose or gain weight N (%)	Not change weight N (%)	N (%)
**Hours per day of TV watching**			
<1 hour per day	2996 (36.1)	2169 (38.4)	5165 (37.0)
1-2 hours per day	2606 (31.4)	1776 (31.4)	4382 (31.4)
>2 hours per day	2699 (32.5)	1705 (30.2)	4404 (31.6)
**Soft drink consumption**			
0 cans a day	2250 (27.3)	1885 (33.5)	4135 (29.8)
>0-2 cans a day	4958 (60.2)	3095 (55.1)	8053 (58.1)
>2 cans a day	1029 (12.5)	640 (11.4)	1669 (12.0)
**Breakfast consumption***			
0-2 days	2027 (24.8)	1089 (19.6)	3116 (22.7)
3-4 days	1884 (23.1)	1225 (22.0)	3109 (22.7)
5 days	4251 (52.1)	3247 (58.4)	7498 (54.6)
**After-school physical activity***			
0-1 days	2134 (25.7)	1570 (27.7)	3704 (26.5)
2-3 days	3082 (37.1)	2101 (37.1)	5183 (37.1)
4-5 days	3101 (37.3)	1989 (35.1)	5090 (36.4)

*Days out of past 5 school days.
