# Supplementary material for: Sizing the association between lifestyle behaviours and fatness in a large, heterogeneous sample of youth of multiple ethnicities from 4 countries
Source: Int J Behav Nutr Phys Act. 2013 Oct 12;10:115. doi: 10.1186/1479-5868-10-115 (PMC3853713; doi:10.1186/1479-5868-10-115)

Relationships between **TV watching** and body composition variables (adjusted for age, sex and ethnicity) by weight-control attempt and among all participants.

Error bars indicate 95% confidence intervals

BMI: P=0.0001 for “weight-control attempt x TV watching” interaction

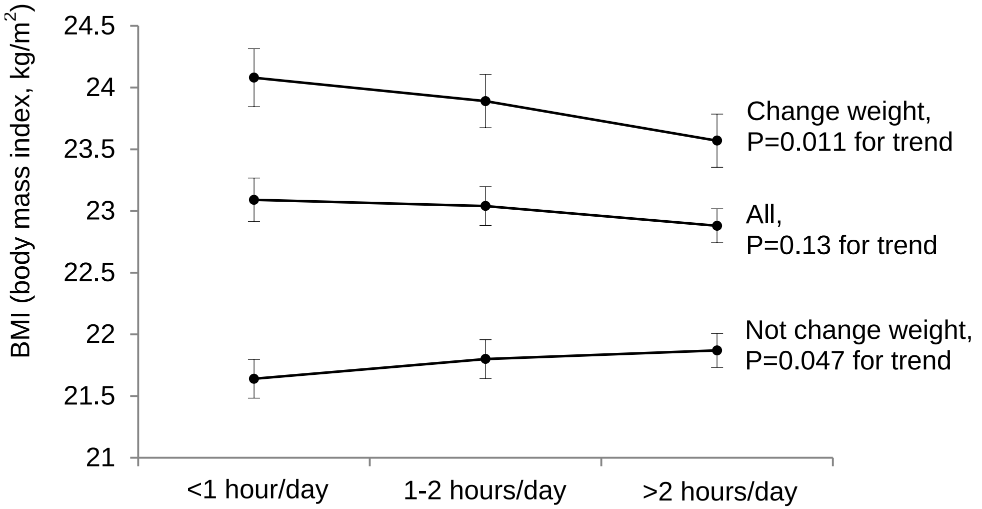

BMIz: P<0.0001 for “weight-control attempt x TV watching” interaction

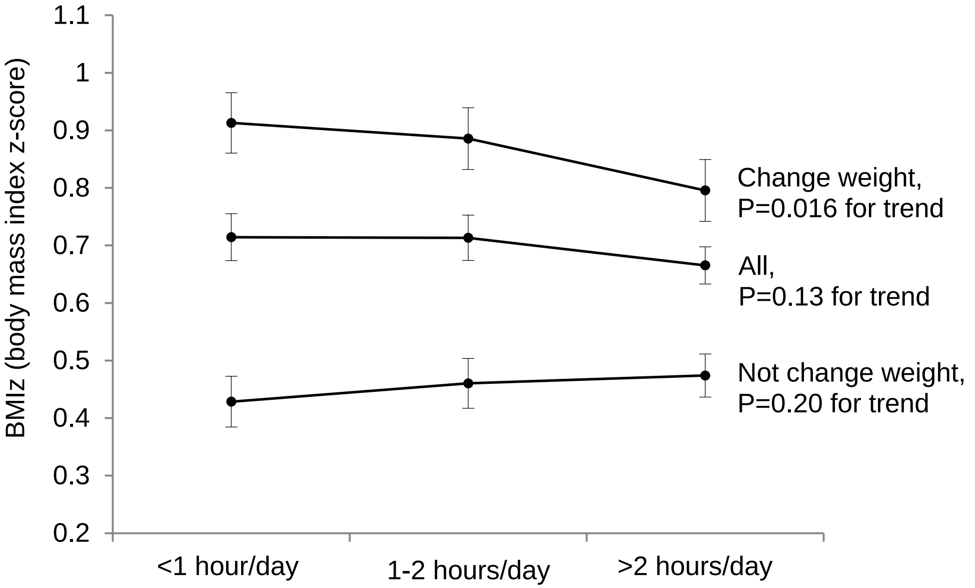

%BF: P=0.0003 for “weight-control attempt x TV watching” interaction

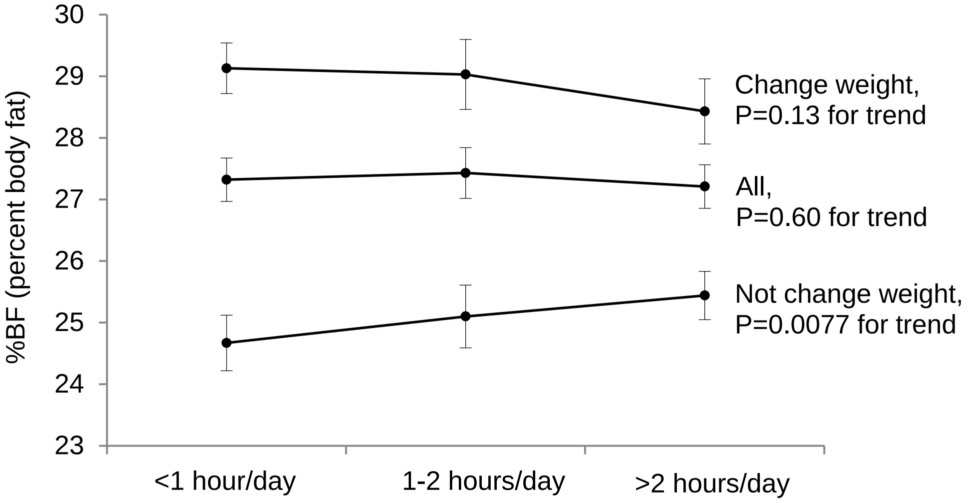

TFM: P=0.0002 for “weight-control attempt x TV watching” interaction

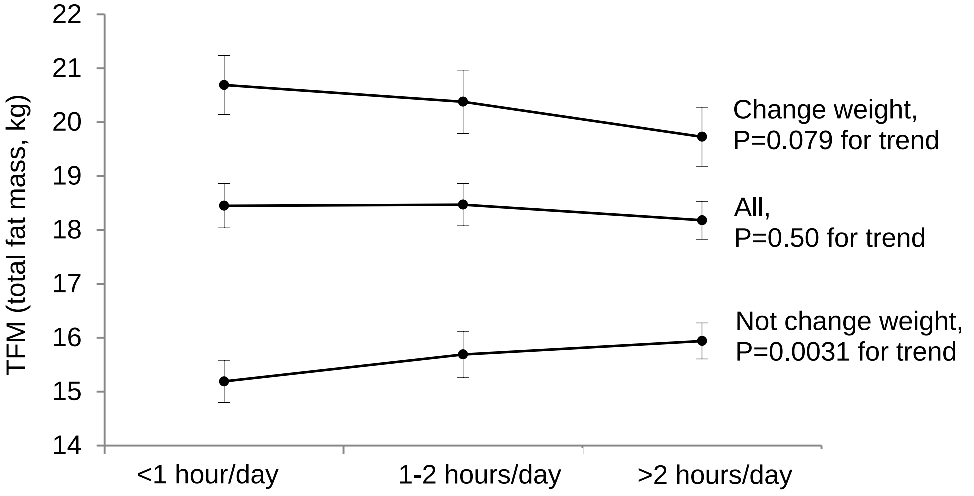

Relationships between **soft drink consumption** and body composition variables (adjusted for age, sex and ethnicity) by weight-control attempt and among all participants. Error bars indicate 95% confidence intervals

BMI: P<0.0001 for "weight-control attempt x consumption" interaction

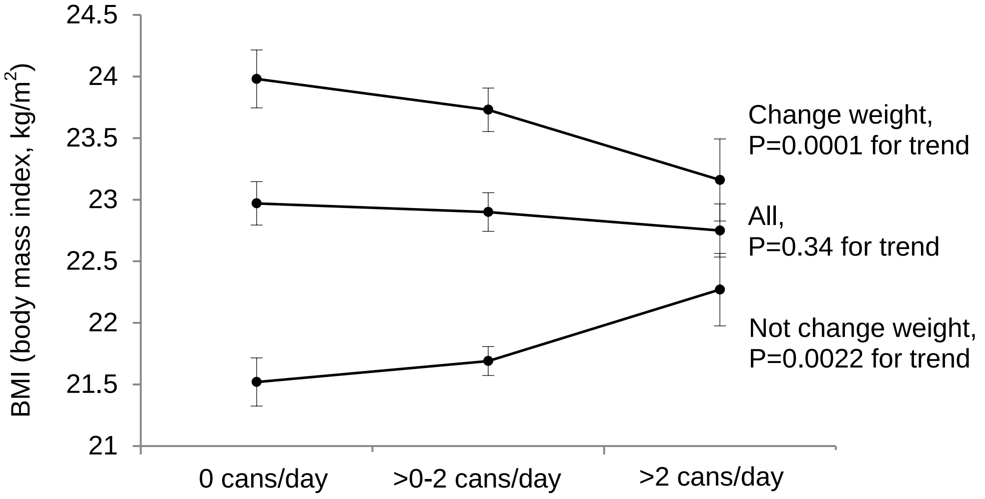

BMIz: P<0.0001 for "weight-control attempt x consumption" interaction

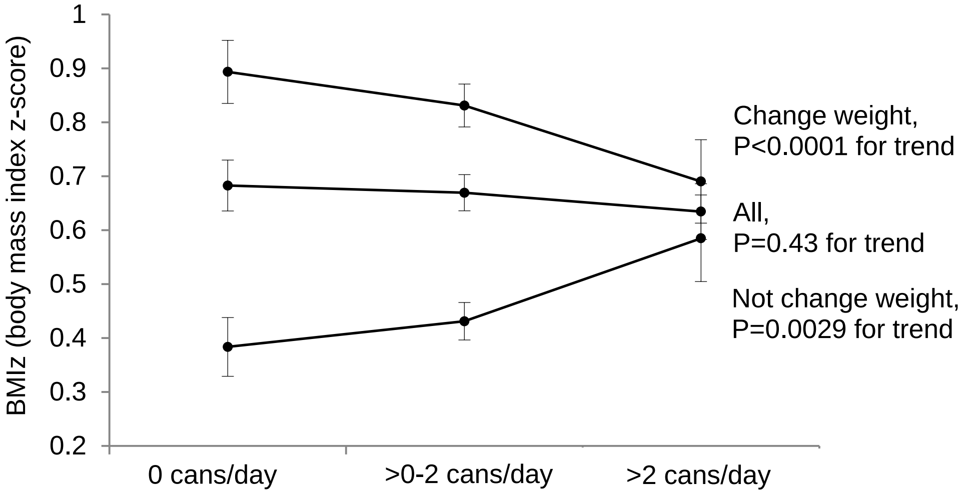

%BF: P<0.0001 for "weight-control attempt x consumption" interaction

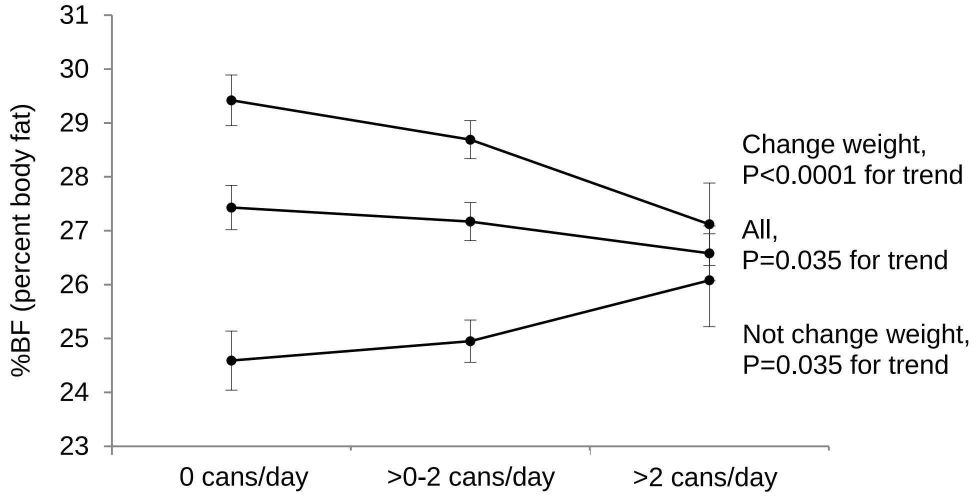

TFM: P<0.0001 for "weight-control attempt x consumption" interaction

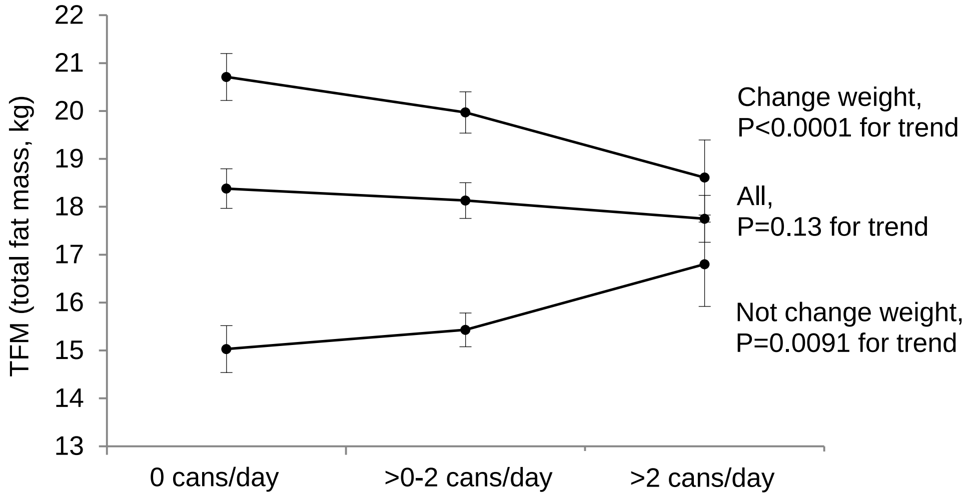

Relationships between **breakfast consumption** and body composition variables (adjusted for age, sex and ethnicity) by weight-control attempt and among all participants. Error bars indicate 95% confidence intervals

BMI: P=0.012 for “weight-control attempt x consumption” interaction

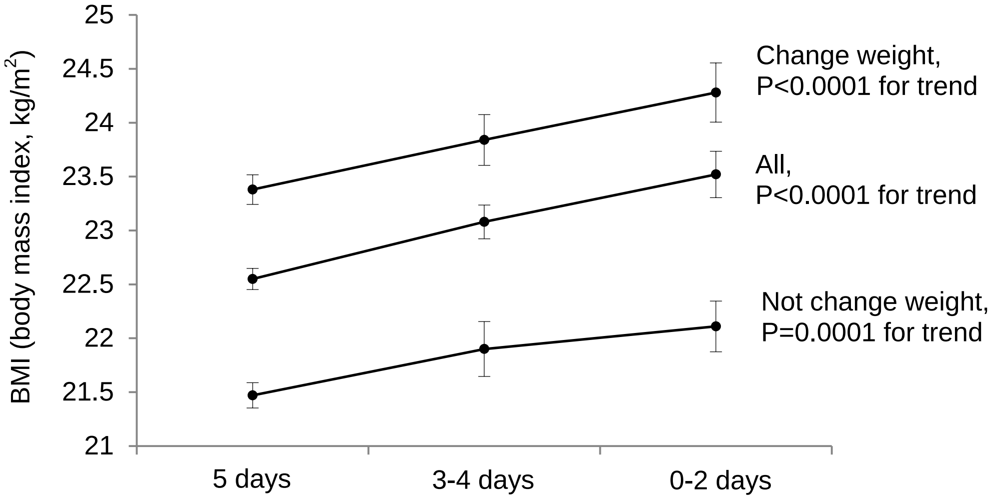

BMIz: P=0.054 for “weight-control attempt x consumption” interaction

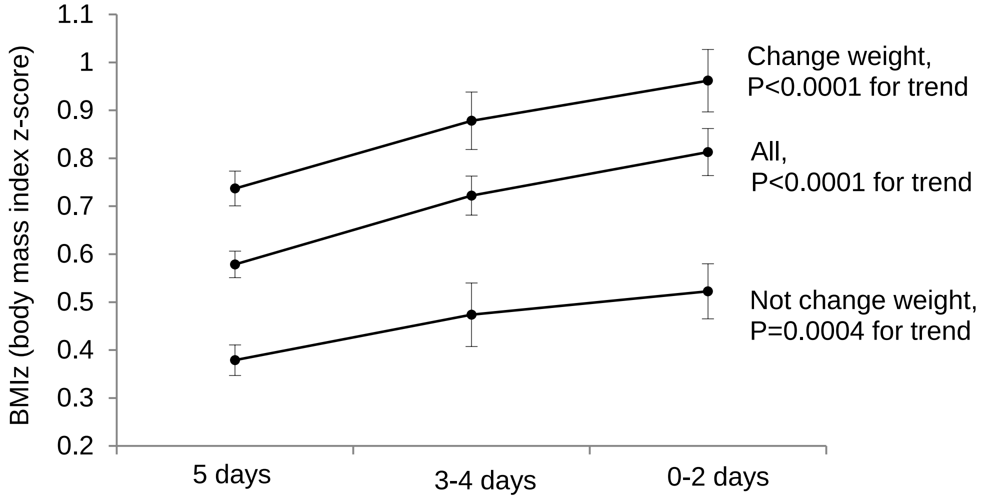

%BF: P=0.023 for “weight-control attempt x consumption” interaction

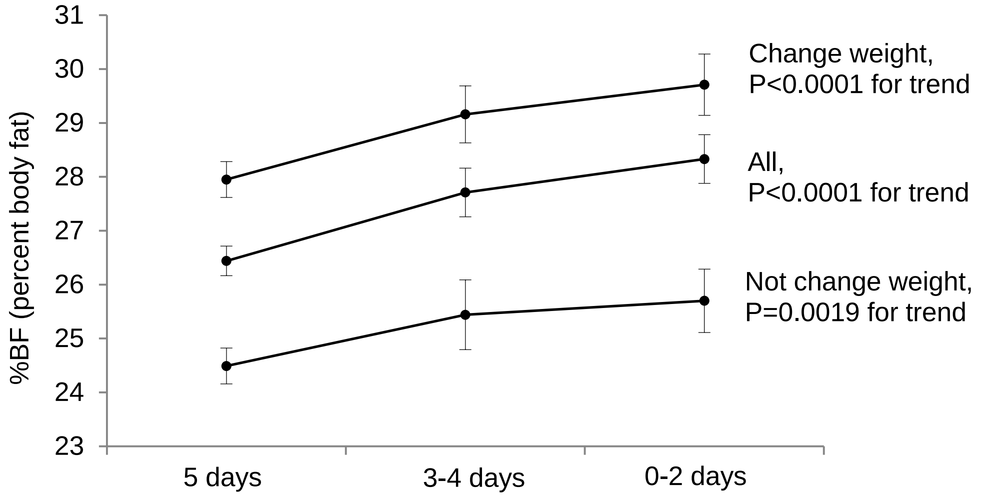

TFM: P=0.0017 for “weight-control attempt x consumption” interaction

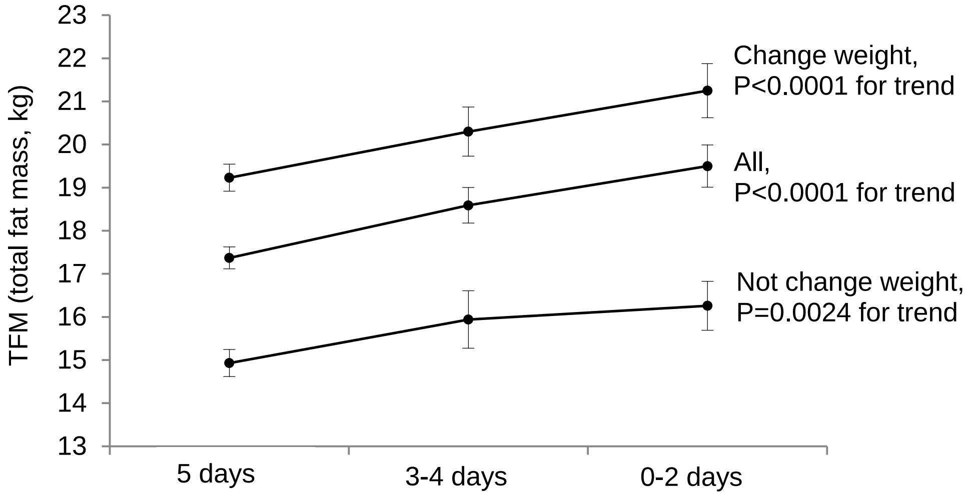

Relationships between **after-school physical activity** and body composition variables (adjusted for age, sex and ethnicity) by weight-control attempt and among all participants. Error bars indicate 95% confidence intervals

BMI: P=0.032 for "weight-control attempt x activity" interaction

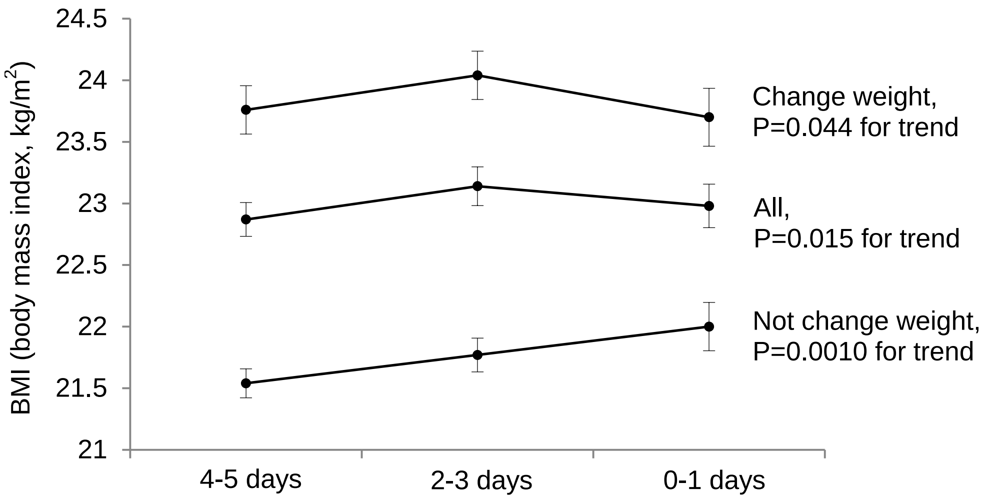

BMIz: P=0.045 for "weight-control attempt x activity" interaction

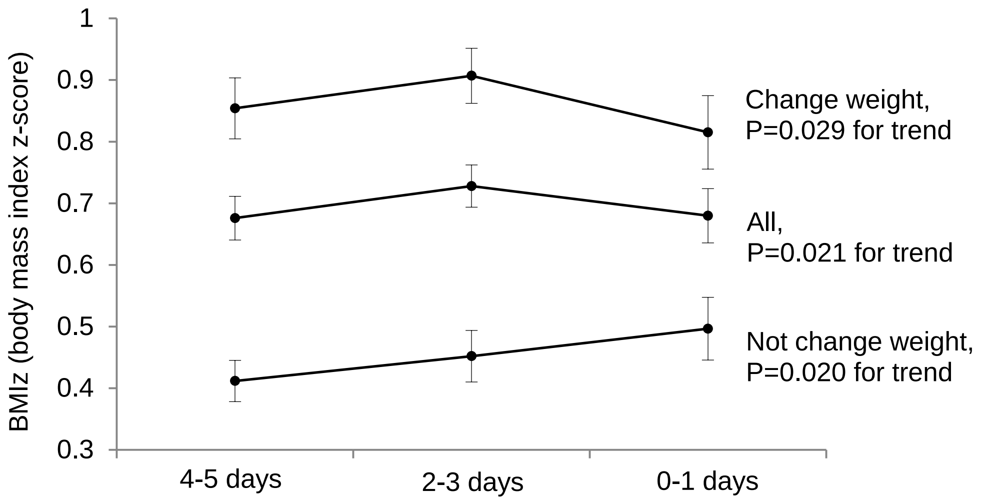

%BF: P=0.0036 for "weight-control attempt x activity" interaction

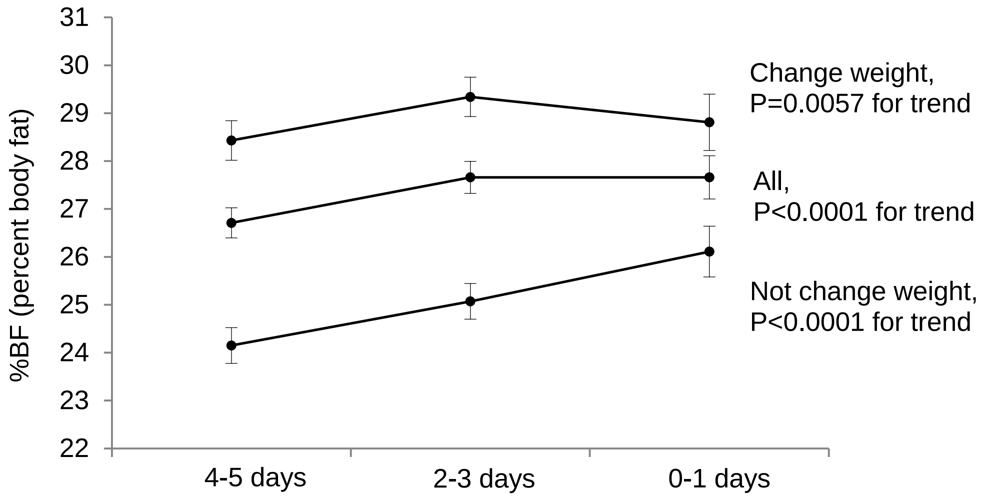

TFM: P=0.008 for "weight-control attempt x activity" interaction

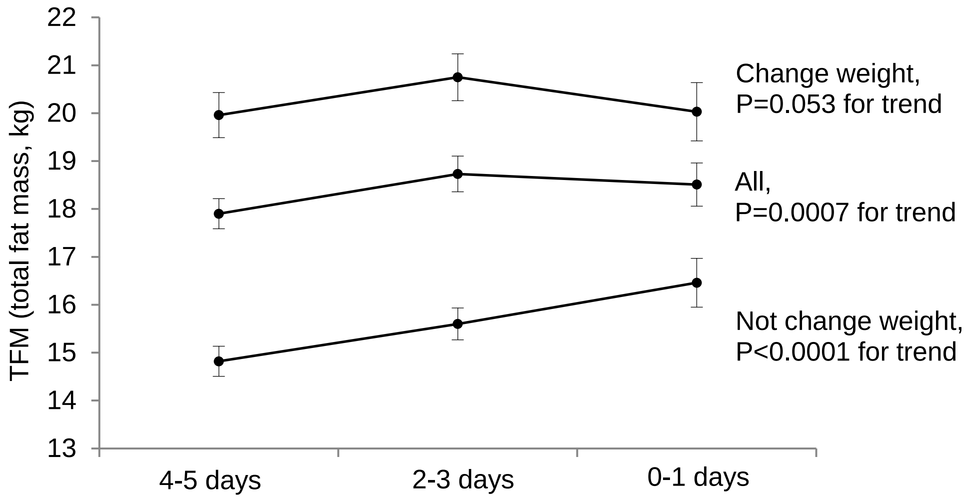

Supplement: Additional file 2 — Relationships between lifestyle and body composition variables (adjusted for age, sex and ethnicity) by weight-control attempt and among all participants. Graphs of lifestyle-fatness associations. [file 1479-5868-10-115-S2.pdf]
